# The 2023 Model Core Content of Disaster Medicine

**DOI:** 10.1017/S1049023X23006556

**Published:** 2023-12

**Authors:** Bryan J. Wexler, Carl Schultz, Paul D. Biddinger, Gregory Ciottone, Angela Cornelius, Robert Fuller, Roxanna Lefort, Andrew Milsten, James Phillips, Ira Nemeth

**Affiliations:** 1. WellSpan Health, WellSpan York Hospital, Department of Emergency Medicine, York, Pennsylvania USA; 2. UC Irvine School of Medicine, Orange, California USA; 3. Harvard Medical School, Harvard University TH Chan School of Public Health, Boston, Massachusetts USA; 4. Beth Israel Deaconess Medical Center, Harvard Medical School, Boston, Massachusetts USA; 5. John Peter Smith Hospital, Fort Worth Emergency Medicine Residency, Fort Worth, Texas USA; 6. University of Connecticut School of Medicine, Farmington, Connecticut USA; 7. Indiana University School of Medicine, Dept of Emergency Medicine, Riley Hospital for Children, Indianapolis, Indiana USA; 8. UMass Chan Medical School, Worcester, Massachusetts USA; 9. The George Washington University Hospital, Department of Emergency Medicine, Washington, DC USA; 10. Henry JN Taub Department of Emergency Medicine, Baylor College of Medicine, Houston, Texas USA

**Keywords:** curriculum, Disaster Medicine, education

## Abstract

**Introduction:**

Disaster Medicine (DM) is the clinical specialty whose expertise includes the care and management of patients and populations outside conventional care protocols. While traditional standards of care assume the availability of adequate resources, DM practitioners operate in situations where resources are not adequate, necessitating a modification in practice. While prior academic efforts have succeeded in developing a list of core disaster competencies for emergency medicine residency programs, international fellowships, and affiliated health care providers, no official standardized curriculum or consensus has yet been published to date for DM fellowship programs based in the United States.

**Study Objective:**

The objective of this work is to define the core curriculum for DM physician fellowships in the United States, drawing consensus among existing DM fellowship directors.

**Methods:**

A panel of DM experts was created from the members of the Council of Disaster Medicine Fellowship Directors. This council is an independent group of DM fellowship directors in the United States that have met annually at the American College of Emergency Physicians (ACEP)’s Scientific Assembly for the last eight years with meeting support from the Disaster Preparedness and Response Committee. Using a modified Delphi technique, the panel members revised and expanded on the existing Society of Academic Emergency Medicine (SAEM) DM fellowship curriculum, with the final draft being ratified by an anonymous vote. Multiple publications were reviewed during the process to ensure all potential topics were identified.

**Results:**

The results of this effort produced the foundational curriculum, the 2023 Model Core Content of Disaster Medicine.

**Conclusion:**

Members from the Council of Disaster Medicine Fellowship Directors have developed the 2023 Model Core Content for Disaster Medicine in the United States. This living document defines the foundational curriculum for DM fellowships, providing the basis of a standardized experience, contributing to the development of a board-certified subspecialty, and informing fellowship directors and DM practitioners of content and topics that may appear on future certification examinations.

## Introduction

Disaster Medicine (DM) is the clinical specialty whose expertise includes the care and management of patients and populations outside conventional care protocols. While traditional standards of care assume the availability of adequate resources, DM practitioners plan for, mitigate against, operate in, and lead recovery from situations where the available resources are not adequate, necessitating modifications to conventional practice. The specialty practices in all environments, from traditional hospital settings to austere locations, and supports the provision of care for all age groups and conditions. It includes administrative leadership of emergency preparedness teams and systems as well as planning and mitigation strategies across the entire disaster cycle. Disaster Medicine also includes expertise in the rational, effective, and ethical adjustment of resource allocation and treatment algorithms during times of resource scarcity to provide the optimal level of health care to the greatest number possible given the circumstances of a disaster or public health emergency.

As recent history demonstrates, disasters resulting from environmental and human activity are growing in complexity, while society’s vulnerabilities to such events are increasing due to a multitude of factors related to the modern world. These events create unique clinical situations that physicians have not anticipated, and for which they are inadequately prepared by traditional medical educational systems to address. Specifically, most training algorithms fail because they were not designed to guide management of the health care requirements of patients when these needs exceed available resources. As an example, the coronavirus disease 2019/COVID-19 pandemic demonstrated how unprepared the medical system is for large-scale medical emergencies, such as a global pandemic, particularly from the standpoint of medical practice outside of established standards of care. Equipment, pharmaceutical, and staff shortages required physicians to make difficult decisions and, in some cases, adopt scarce resource allocation protocols.

The specialty of DM incorporates knowledge and skills from multiple disparate fields including, but not limited to, emergency medicine, prehospital medicine, public health crisis response, critical care, primary care and preventative medicine, ethics, health equity, risk communication, and emergency management, among others. Within DM, elements from all these fields converge into a cohesive practice aimed at reducing mortality and morbidity by optimizing resource use in the event of a disaster. The scope of this multidisciplinary expertise and its requisite associated clinical and administrative skills defines the practice of DM as unique among existing medical specialties.

When applied to health care specifically, a disaster implies a severe imbalance in patient needs and available resources. The ability to effectively and efficiently utilize limited assets to provide maximal benefit for an affected population requires a keen understanding of resource utilization and population health. In response to such increasingly austere environments, physicians with DM specialty training are needed to transition patient care efficiently from conventional operations to progressively limited resource utilization and treatment strategies. Individuals possessing this training can plan, implement, and assess appropriate patient management in the highly variable situations and locations in which the provision of medical care may be required. Prior to disaster-related events, DM specialists guide implementation of preventative and mitigating actions to limit the impact of a future disaster, optimizing the ability to continue providing patients the conventional standard of care. However, if a patient surge is so vast, or a reduction in available resources so profound, that existing standards of care are not possible, specialists in DM are uniquely qualified to ethically guide stakeholders and leaders through the allocation of constrained resources during a crisis.

Disaster Medicine’s unique combination of clinical, administrative, and ethical expertise, in addition to its requisite skills focused on the delivery of medical care in nontraditional practice environments, is not found in any current medical specialty. Though selected elements may be found in, and are drawn from, other defined areas of medicine, no other subspecialty holistically focuses on such expertise. For example, the existing subspecialty of Emergency Medical Services (EMS) addresses mass-casualty response in a very limited capacity and viewed through the lens of the prehospital setting only.^1^ Toxicology practitioners obtain expertise in treating chemical poisonings, but the core content does not address planning, preparation, or response to a mass-casualty nerve agent attack.^2^ While prior academic efforts have succeeded in developing a list of core disaster competencies for emergency medicine residency programs, international fellowships, and affiliated health care providers, no official standardized curriculum or consensus has yet been published to date for DM fellowship programs based in the United States.^3–5^

## Methods

The initial outline of the 2023 Model Core Content of Disaster Medicine was developed with support from a task force of DM physicians convened by the American College of Emergency Physicians (ACEP; Irving, Texas USA) Disaster Preparedness and Response Committee. This group incorporated the previous DM curriculum used by the Society of Academic Emergency Medicine (SAEM; Des Plaines, Illinois USA), aimed at standardizing SAEM-approved DM fellowships.^6^ The draft curriculum was presented to the Council of Disaster Medicine Fellowship Directors. This council of active DM fellowship directors was formed in 2015, meeting annually as an interim guiding body in the absence of formal sub-specialization recognition. The presented draft curriculum was updated by drawing from existing individual DM fellowship curricula provided by the active fellowship directors on the Council of Disaster Medicine Fellowship Directors. All topics were cross-referenced with the Accreditation Council on Graduate Medical Education’s (ACGME; Chicago, Illinois USA) six core competencies for the practice of medicine.^7^ After a new draft was composed by the task force, using a modified Delphi technique, the DM fellowship directors on the Council of Disaster Medicine Fellowship Directors individually provided additional review and input on all aspects, incorporating material from existing DM textbooks previously absent from the curriculum.^8,9^ The option to provide anonymous feedback was available. The proposed curriculum was revised over three additional iterations to create a new, more comprehensive document that was subsequently validated and approved by the voting members of the council, defined as having an active DM fellowship, in an anonymous, unanimous vote with none voting against the motion.

## Results

To address the need for a standardized core curriculum supporting the training of DM practitioners in the United States, the Council of Disaster Medicine Fellowship Directors created a new curriculum. This model is recommended as the foundational course of study for physicians training in formal United States DM fellowship programs (Table 1).

**Table 1.** The Model Core Content of Disaster Medicine

	ACGME and ABMS Core Competencies					
	Patient Care	Medical Knowledge	Practice-Based Learning	Professionalism	Interpersonal Skills	Systems-Based Practice
**Principles Of Disaster Medicine (1)**	X	X	X			X
Conventional Standards Of Care (1.1)	X	X	X			X
Contingency Standards of Care (1.2)	X	X	X			X
Crisis Standards Of Care (1.3)	X	X	X	X	X	X
Disaster Triage Concepts (1.4)	X	X	X	X	X	X
Scarce Resource Allocation Protocols (1.5)	X	X	X	X	X	X
**Medical Oversight of Emergency Management Systems (2)**	X	X	X	X	X	X
The Disaster Cycle (2.1)				X		X
Evolution of Emergency Management (2.2)				X		X
Local Disaster Response (2.3)				X	X	X
National Disaster Response (2.4)				X	X	X
Disaster Medical Assistance Teams (DMAT) (2.4.1)				X	X	X
National Response Framework (2.5)				X	X	X
National Incident Management System (2.5.1)				X	X	X
ICS Basics (2.5.2)				X	X	X
Hospital Preparedness Program (2.5.3)				X	X	X
Strategic National Stockpile (2.5.4)	X					X
National Disaster Management System (2.5.5)				X	X	X
International Systems (2.6)						X
UN Cluster System (2.6.1)						X
Emergency Medical Teams & World Health Organization (2.6.2)						X
International Search and Rescue Advisory Group (INSARAG) (2.6.3)						X
Nongovernmental Organizations (NGO) (2.7)						X
Exercise Design and Evaluation (2.8)			X	X		X
**Public Health and Disaster Medicine (3)**	X	X		X		X
Role of Public Health Agencies in Disaster Medicine (3.1)						X
Public Health Surveillance (3.2)		X	X			X
Needs Assessments (3.3)	X	X				X
Sphere Standards; Water, Sanitation, and Hygiene (WASH) (3.3.1)	X	X				
Complex Public Health Emergencies (3.4)	X	X	X		X	X
Displaced Populations (3.4.1)	X	X			X	X
Medical Care for Refugee Populations (3.4.2)	X	X			X	X
Climate Change and Disaster Medicine (3.5)		X				
Vaccine and Pharmaceutical Distribution (3.6)	X	X		X	X	X
Quarantine/Isolation (3.7)		X		X	X	X
**Health Care Disaster Preparedness (4)**	X	X	X	X	X	X
Hazard Vulnerability Analysis (4.1)		X	X			X
Drill and Exercise Design (4.1.1)						
Hospital Incident Command Systems (4.2)				X	X	X
Emergency Operations Plans for the Health Care Environment (4.3)		X				X
Command Center Operations (4.4)				X	X	X
Health Care Coalitions and Community Integration (4.5)				X	X	X
Information Management/Communications (4.6)				X	X	X
Medical Surge Capacity (4.7)	X	X				X
Medical Surge Capability (4.8)	X	X				X
Mass-Casualty Incidents (4.9)	X	X				X
**Disaster Preparedness and Resiliency (5)**	X	X	X	X	X	X
Personal Preparedness (5.1)				X		X
Organizational Preparedness and Resiliency (5.2)	X	X	X	X	X	X
Business Continuity (5.2.1)						
Hospital Preparedness (5.3)	X	X	X	X	X	X
Community Preparedness and Resiliency (5.4)		X	X	X	X	X
National Preparedness (5.5)				X		X
Rehabilitation and Reconstruction (5.6)						X
**Operations and Logistics (6)**	X	X	X	X	X	X
Field Operations and Logistics (6.1)	X		X			X
Mass-Casualty Care in the Field (6.2)	X		X			
Field Disaster Triage (6.3)	X	X				X
Field Stabilization, Treatment, and Transport (6.4)	X	X				
Disaster Operations (6.5)						X
Decontamination in the Field (6.6)	X		X			
Volunteer Management (6.7)				X	X	X
Operational Continuity (6.8)						X
Care of Animals (6.9)			X			
Alternative Care Sites (6.10)						X
Mass-Fatality and Mortuary Care (6.11)		X		X	X	X
**Psychological Aspects of Disaster Medicine (7)**	X	X	X	X	X	X
Psychological Effects and Trauma of Disaster (7.1)	X	X			X	X
Psychological First Aid (7.2)	X	X	X		X	
Personal Mental Resiliency (7.3)		X	X	X	X	
**Ethical and Legal Issues in Disaster Medicine (8)**	X			X	X	X
Ethics of Disaster Medicine (8.1)	X			X	X	X
Liability in Disaster Response (8.2)				X	X	X
Disaster Finance (8.3)						X
Stafford Act (8.3.1)						X
Vulnerable Populations (8.4)						X
**Prehospital Disaster Medicine (9)**	X	X	X			X
EMS Disaster Operations (9.1)	X	X				X
Transportation Disasters (9.1.1)		X				
Search and rescue (9.1.2)	X	X				
Tactical EMS (9.1.3)			X			X
Active Threats (9.2)	X		X			X
Care Under Fire (9.2.1)	X		X			
Scene Safety and Security in the Field (9.3)	X	X				X
Fireground Safety (9.3.1)						X
Structural Collapse (9.3.2)	X	X				
Vehicle Extraction (9.3.3)	X	X				
**Chemical, Biological, Radiological, Nuclear, and Explosive (CBRNE) (10)**	X	X	X	X	X	X
Chemical Agents (10.1)	X	X				
Recognition and Clinical Treatment (10.1.1)	X	X				
Blister Agents (10.1.1.1)	X	X				
Lewisite (10.1.1.1.1)	X	X				
Mustard (10.1.1.1.2)	X	X				
Choking Agents (10.1.1.2)	X	X				
Anhydrous Ammonia (10.1.1.2.1)	X	X				
Chlorine (10.1.1.2.2)	X	X				
Phosgene (10.1.1.2.3)	X	X				
Asphyxiant Agents (10.1.1.3)	X	X				
Cyanide (10.1.1.3.1)	X	X				
Nerve Agents (10.1.1.4)	X	X				
MCI Triage and Considerations for Chemical Agents (10.1.2)	X	X		X	X	
Pediatrics and Chemical Exposure (10.1.3)	X	X				
Chemical Safety (10.1.4)						X
Decontamination (10.1.4.1)	X	X	X			
Biological Disasters (10.2)	X	X	X	X	X	X
Category A Bioterrorism Agents (10.2.1)	X	X				
Anthrax (10.2.1.1)	X	X				
Botulism (10.2.1.2)	X	X				
Smallpox (10.2.1.3)	X	X				
Tularemia (10.2.1.4)	X	X				
Viral Hemorrhagic Fevers (10.2.1.5)	X	X				
Yersinia Pestis (10.2.1.6)	X	X				
Clinical Diagnosis and Treatment (10.2.2)	X	X				
MCI Triage and Considerations for Biological Agents (10.2.3)	X	X	X	X	X	
Biological Safety (10.2.4)						X
Epidemiologic and Medical Countermeasures (10.2.5)	X	X				
Radiation/Nuclear Events (10.3)	X	X	X	X	X	X
Acute Radiation Syndrome (10.3.1)	X	X				
Timing of Medical and Surgical Interventions (10.3.2)	X	X				
Contamination and Irradiation (10.3.3)	X	X				
Decontamination (10.3.3.1)	X	X	X			
Medical Countermeasures for Radiation Contamination (10.3.4)	X	X				
MCI Triage and Considerations for Radioactive/Nuclear Events (10.3.5)	X	X	X	X	X	
Pandemics/Emerging Infectious Diseases (10.4)	X	X	X	X	X	X
Epidemiology (10.4.1)		X				X
Mass Care During Pandemics (10.4.2)	X	X		X	X	X
Medical Countermeasures (10.4.3)	X	X				X
Pandemic Triage (10.4.4)	X	X	X	X	X	
Hazardous Materials (HAZMAT) (10.5)		X	X			
Personal Protective Equipment (PPE) (10.5.1)		X	X			
Blast Injuries (10.6)	X	X				
Crush Injuries (10.7)	X	X				
Burns (10.8)	X	X	X			
Mass Burn Care (10.8.1)	X	X	X			
**Mass Care and Environmental Disasters (11)**	X	X				X
Climate Change (11.1)						X
Drought (11.2)						X
Earthquakes (11.3)	X	X				
Flooding (11.4)	X	X				
Heat Emergencies (11.5)	X	X				
Hurricanes/Cyclones/Typhoons (11.6)	X	X				X
Tornadoes (11.7)	X	X				
Volcanic Eruptions (11.8)	X	X				
Wildfires (11.9)						X
Winter Storms (11.10)	X	X				
**Mass-Gathering Medicine (12)**	X	X		X	X	X
Mass Gatherings (12.1)		X				X
Event Medicine Planning (12.1.1)	X			X		X
Stampede Injuries (12.1.2)	X					
Civil Unrest (12.2)				X	X	X
**Communications (13)**				X	X	
Crisis and Emergency Risk Communication (13.1)			X	X	X	X
Media Engagement (13.1.1)				X	X	
Communication Systems and Informatics (13.2)						X
Social Media and Disasters (13.3)				X	X	X
**Technology and Disaster Medicine (14)**	X	X	X			X
Technological Disasters (14.1)						X
Utility Failure (14.1.1)						X
Informatics (14.2)						X
Electronic Health Record Compromise (14.2.1)		X				X
Disaster Modeling and Simulation (14.3)			X			
Crisis Mapping (14.4)						X
Patient Tracking (14.5)	X					X
Telemedicine (14.6)	X		X			X
Ultrasound (14.7)	X	X	X			
**Disaster Medicine Research (15)**						
Journal Club (15.1)		X	X			
Research basics (15.2)		X				X

Abbreviations: ACGME, Accreditation Council on Graduate Medical Education; ABMS, American Board of Medical Specialties; ICS, Incident Command Systems; EMS, Emergency Medical Services; MCI, Mass-Casualty Incident.

## Discussion

The 2023 Model Core Content of Disaster Medicine approaches each domain within DM through the lens of the disaster medical specialist, drawing from best practices to provide the best possible care under adverse circumstances. The resultant curriculum is a living document that is intended to be reviewed and updated periodically to address the evolving field of DM. It is not expected that fellowships will be teaching the curriculum exclusively. Some programs may have special areas of focus or interests that they wish to highlight in their fellowship training. However, all programs are expected to utilize the curriculum to provide a consistent and comprehensive knowledge base for graduating fellows.

Official recognition of DM as a board-certified subspecialty will lead to the development of training programs producing specialists with a known and consistent professional expertise. Disaster Medicine experts will lead efforts to provide education, training, comprehensive emergency operational plans, and disaster medical direction prior to and during a crisis. This Model Core Content document outlines the comprehensive knowledge base and skills DM specialists are expected to master and that may appear on future certification examinations.

## Limitations

The Model Core Content attempts to minimize overlap among its topics; however, some areas are integrated and may be covered in different ways in other sections. It is expected the content will continue to evolve as the field of DM develops and additional needs and informational gaps are identified. The 2023 Model Core Content of Disaster Medicine defines the foundational curriculum for DM physician fellowships in the United States, provides the basis of a standardized experience, and contributes to the future development of a board-certified subspecialty. However, the recommended content may not necessarily translate to other educational systems external to the United States.

Additional limitations reside within the Delphi methodology itself. In this work, the expert panel included the directors responsible for the education of fellows. However, the panel was limited to the Council of Disaster Medicine Fellowship Directors and there is potential for additional views not represented, such as from DM fellowships whose existence was unknown to the council at the time of the curriculum development. Furthermore, while anonymous feedback in each iteration was available, some chose to make their suggestions public, potentially influencing other panelists as open discussion was also utilized frequently between panelists during evaluation between iterations. However, given the final anonymous ratification, the authors believe this potential influence was not significant.

## Conclusion

The 2023 Model Core Content of Disaster Medicine is a living document that defines the foundational curriculum for DM fellowships, providing the basis of a standardized experience, contributing to the development of a board-certified subspecialty, and informing fellowship directors and DM practitioners of content and topics that may appear on future certification examinations.
